# Structural basis of inhibition of lipid-linked oligosaccharide flippase PglK by a conformational nanobody

**DOI:** 10.1038/srep46641

**Published:** 2017-04-19

**Authors:** Camilo Perez, Martin Köhler, Daniel Janser, Els Pardon, Jan Steyaert, Renato Zenobi, Kaspar P. Locher

**Affiliations:** 1 Department of Biology, Institute of Molecular Biology and Biophysics, ETH Zürich, CH-8093 Zürich, Switzerland; 2 Department of Chemistry and Applied Biosciences, ETH Zürich, CH-8093 Zürich, Switzerland; 3 VIB Center for Structural Biology, VIB, 1050 Brussels, Belgium; 4 Structural Biology Brussels, Vrije Universiteit Brussel, 1050 Brussels, Belgium

## Abstract

PglK is an ABC transporter that flips a lipid-linked oligosaccharide (LLO) that serves as a donor in protein N-glycosylation. Previous structures revealed two inward-facing conformations, both with very large separations of the nucleotide binding domains (NBDs), and a closed, ADP-bound state that featured an occluded cavity. To investigate additional states, we developed conformation-sensitive, single-domain camelid nanobodies (Nb) and studied their effect on PglK activity. Biochemical, structural, and mass spectrometric analyses revealed that one inhibitory Nb binds as a single copy to homodimeric PglK. The co-crystal structure of this Nb and ADP-bound PglK revealed a new, narrowly inward-open conformation. Rather than inducing asymmetry in the PglK homodimer, the binding of one Nb results in steric constraints that prevent a second Nb to access the symmetry-related site in PglK. The Nb performed its inhibitory role by a “sticky-doorstop” mechanism, where inhibition of ATP hydrolysis and LLO flipping activity occurs due to impaired closing of the NBD interface, which prevents PglK from converting to an outward-open conformation. This inhibitory mode suggests tight conformational coupling between the ATPase sites, which may apply to other ABC transporters.

ABC transporters are a ubiquitous family of membrane proteins with diverse functions in biology. PglK is a homodimeric ABC transporter with an essential role in the protein N-glycosylation machinery of the pathogenic bacterium *Campylobacter jejuni*^1^. It flips the complex lipid-linked oligosaccharide (LLO) GlcGalNac_5_Bac-PP-undecaprenyl, which is assembled through the sequential, catalytic activities of the glycosyltransferases PglC, PglA, PglJ, PglH and PglI^2^, from the cytoplasmic side of the membrane into the periplasm^1^, where the oligosaccharyltransferase PglB catalyzes the transfer of the glycan to acceptor proteins^3^. A homologous process occurs in eukaryotes, where the LLO Man_5_GlcNAc_2_-PP-dolichol is flipped from the cytosolic side to the luminal side of the endoplasmic reticulum and, after extension of the glycan, is transferred to acceptor proteins by oligosaccharyltransferase (OST), a hetero-oligomeric membrane protein complex^4^,^5^.

We have previously reported structures of PglK that provided insight into, and suggested, an unusual, “outward-only” LLO translocation mechanism^6^, whereby only outward-facing conformations are required to provide a translocation pathway for the oligosaccharide-pyrophosphate head-group of LLO, whereas the undecaprenyl tail remains embedded in the lipid bilayer. A more detailed understanding of the challenging flipping mechanism requires structural insight into as many states as possible. We therefore sought to use Nbs to trap PglK in additional conformations for high-resolution structure determination. Camelids produce a unique class of antibody molecules devoid of light chains^7^. A nanobody (Nb) is the minimal antigen-binding domain of the camelid heavy chain antibody^8^, their use has provided remarkable progress in stabilizing conformers of other transporters and G-protein coupled receptors^9^,^10^,^11^,^12^. Studies on modes of inhibition of ABC transporters, including inhibition by nanobodies (Nbs), are of general interest because of the importance of these membrane proteins in many biological transport processes^13^,^14^,^15^. Small molecule inhibitors targeting diverse ABC transporters have been reported, but usually target substrate-binding sites, where they compete with substrate binding^15^,^16^. In contrast, antibodies and Nbs can lock certain conformations or states by binding at sites other than the substrate-binding pockets^9^,^17^,^18^,^19^. However, only few studies have reported structures of ABC exporters bound to Nbs or antibodies^17^,^18^,^20^.

Here we produced conformational nanobodies against PglK and determined the structure of one inhibitory nanobody bound to PglK. The structure revealed a novel, narrowly inward-facing state with bound ADP, where both NBDs are much closer than in previous wide-open apo-inward facing structures^6^. This is the fourth conformation of PglK visualized at high resolution. Our crystal structure, together with results from complementing methods, revealed a “sticky-doorstop” inhibitory mechanism, where the Nb prevents the transition to closed NBDs sandwich dimer conformations and the binding of a second Nb molecule. This inhibitory mechanism also has implications for the LLO flipping mechanism and points towards a tight conformational coupling between the two ATP hydrolysis sites.

## Results and Discussion

### Nanobodies screening

To generate conformational nanobodies, we immunized a llama with purified PglK reconstituted in proteoliposomes. After generating a Nb library and screening, we identified 26 distinct families based on sequence alignments. Pull-down assays showed that representative Nbs from each family co-elute with PglK, indicating the formation of stable PglK-Nb complexes (Supplementary Figure 1). As negative control we have used NbPglB, a control Nb specific for bacterial oligosaccharyltransferase PglB (data not shown).

We were particularly interested in identifying inhibitory nanobodies because only these have the potential to lock certain states of the target transporter. Therefore, purified PglK was incubated with representative Nbs from each family. We found that the Nbs have varying degrees of effect on ATPase activity, with Nbs 84, 87, 93 and 97 strongly inhibiting PglK in detergent solution (Supplementary Figure 2A). When PglK was reconstituted in proteoliposomes, Nbs 84, 87, 93 and 97 still inhibited ATPase activity, but to a lower degree than in detergent (Supplementary Figure 2B), which may reflect differences in the population of PglK conformers in detergent compared to proteoliposomes.

### Crystallization of PglK-E510Q in complex with Nb87

**Figure 1:**
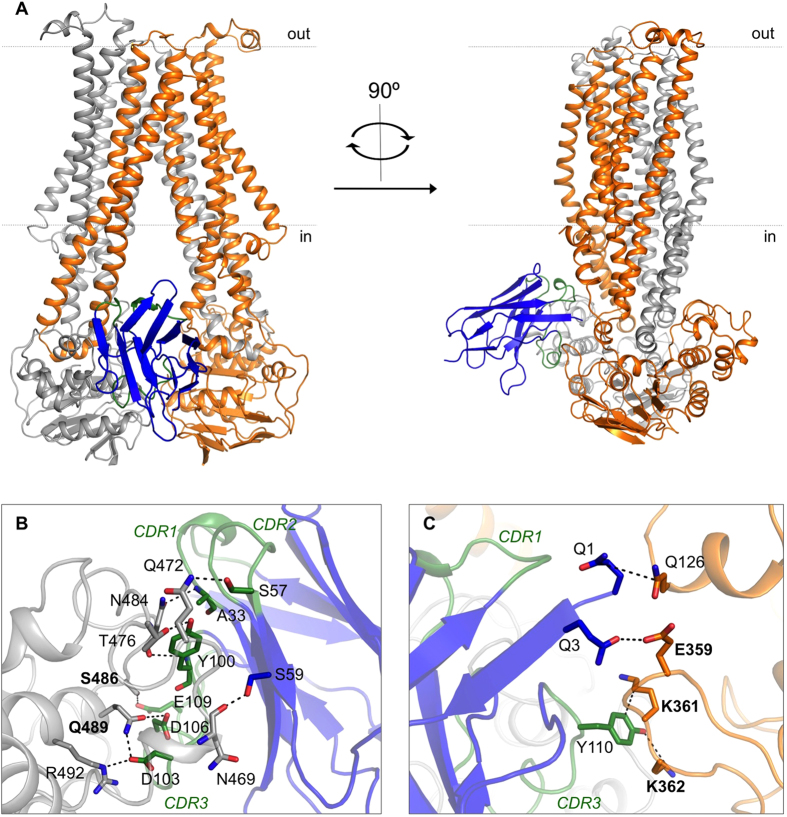
(**A**) Structure of PglK in complex with inhibitory nanobody Nb87. PglK subunits are shown as gray and orange ribbons. The backbone of Nb87 is shown in blue, with CDRs in green. Dotted lines denote membrane boundaries. (**B** and **C)** Details of interactions at the interface of Nb87 with the NBDs of PglK. Dotted lines denote hydrogen bonds or salt bridges.

We attempted co-crystallization of PglK with each of the four inhibitory Nbs, and obtained well-diffracting crystals with Nb87. The PglK mutant E510Q was used for crystallization experiments due to its higher expression yield and stability compared to PglK wild type (WT). We have previously reported that this mutant retains ATPase and flipping activity, albeit with slower rates^6^. PglK-E510Q was purified (Supplementary Figure 3), followed by incubation with Nb87. Intriguingly, crystals were only observed when ADP and MgCl_2_ were present. After extensive optimization of crystallization conditions, constructs, additives and cryo-protection, the structure of the complex could be determined at 3.9 Å resolution (Fig. 1, Supplementary Figures 4 and 5; and Extended Data Table 1). The electron density map was of sufficient quality to trace most of the side chains of PglK and Nb87 with high confidence (Supplementary Figures 4 and 5). The crystal packing shows that external loops and NBDs of PglK, and loops of Nb87, formed lattice contacts (Supplementary Figure 6). Notably, one of the external helices (EHs) of PglK is involved in a crystal contact. Consequently, the conformations of the two EHs in the PglK homodimer are distinct (Supplementary Figure 6).

The electron density map shows clear density for a single Nb87 bound to the PglK homodimer (Supplementary Figures 4 and 5). Nb87 binds PglK close to the membrane boundary (Fig. 1A) and contacts both NBDs, thus fixing the NBD distance within PglK. The three complementarity-determining regions (CDRs) of Nb87 comprise one short loop (CDR2: I51-T58) and two long ones (CDR1: S25-M34; CDR3: A97-S112). All three CDRs contribute to the interface with NBD1 (colored gray), whereas only CDR3 and the N-terminus of Nb87 contribute to the interface with NBD2 (colored orange) (Fig. 1B and C). Most of the Nb87-PglK interactions are hydrophilic in nature, involving not only side chains but also backbone atoms. The interactions with NBD1 involve residues from the catalytically important signature (or LSGGQ) motif, specifically with the side chains of S486 and Q489 (Fig. 1B). Contacts with the NBD2 involve interactions with the side chains of E359, K361 and K362, from the functionally important A-loop that contributes to the binding of the adenine moiety of ATP. There is also a contact between Nb87 and Q126 from one of the coupling helices of PglK (Fig. 1C)^21^,^22^.

### Narrow inward-facing conformation

**Figure 2:**
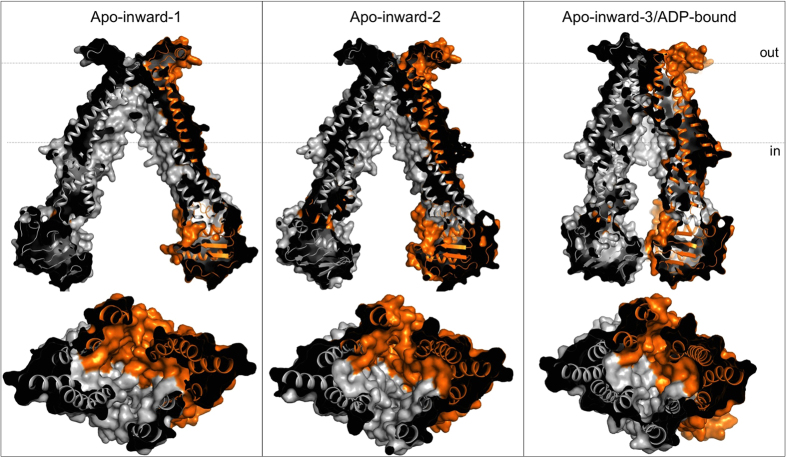
Comparison of central cavities and NBD separation in inward-facing structures of PglK. The bottom panel represents a slice view from the cytoplasm. Dotted lines denote membrane boundaries. Apo-inward-1 (PDB code 5C78); Apo-inward-2 (PDB code 5C76); Apo-inward-3/ADP-bound, this study (PDB code 5NBD).

The Nb87-trapped PglK structure reveals a novel, narrowly inward-facing conformation, with only few contacts between the NBDs. This is distinct from the full separation observed in the two previously reported inward-facing structures (Fig. 2), but also from the almost completely closed sandwich dimer observed in the ADP-bound outward-occluded state reported^6^. The effective volume of the central cavity of Nb87-bound PglK is reduced by ~50% when compared to the apo-inward-1 structure, and by ~40% when compared to the apo-inward-2 state (Fig. 2)^6^,^23^. The NBD and transmembrane domain (TMD) opening and angles are similar to the observed conformation of cryo-electron microscopy structures of heterodimeric TmrAB^20^ and of human ABCB1^18^, both of which had no nucleotides bound, and the structures of heterodimeric TM287/288 with AMP-PNP bound^24^, and the human ABCB10 with AMP-PCP bound^25^. Even though the NBDs of Nb87-bound PglK are not forming a closed sandwich dimer, two weak positive peaks in the Fo-Fc map below the P-loops (Walker A motifs) were observed (Supplementary Figure 7A). Since this location corresponds to the binding site of the di-phosphate and tri-phosphate groups of ADP and ATP, respectively, we interpret these peaks as indicating partially occupied ADP molecules bound to the ATPase sites, and thus interpret our Nb87-bound PglK structure as an inhibited, apo-inward/ADP-bound state^21^,^22^, nevertheless, nucleotide exchange may occur in this state.

We have previously described eight functionally relevant arginine residues (R86, R260, R302, R309) in the central cavity of outward-open PglK, where they probably interact with the pyrophosphate moiety of transport substrate, LLO^6^. These arginines were found buried and forming salt-bridges and cation-π interactions in the apo-inward-1 and apo-inward-2 states. Despite the narrowing of the cavity and the contact of the NBDs, the architecture of the central cavity of Nb87-bound PglK has not changed significantly and the functionally important arginines are still engaged in the same interactions as in the two widely inward-open states (Supplementary Figure 7B). This suggests that they will only become accessible upon conversion of PglK to an outward-facing state.

### PglK-Nb87 stoichiometry and inhibitory mechanism

**Figure 3:**
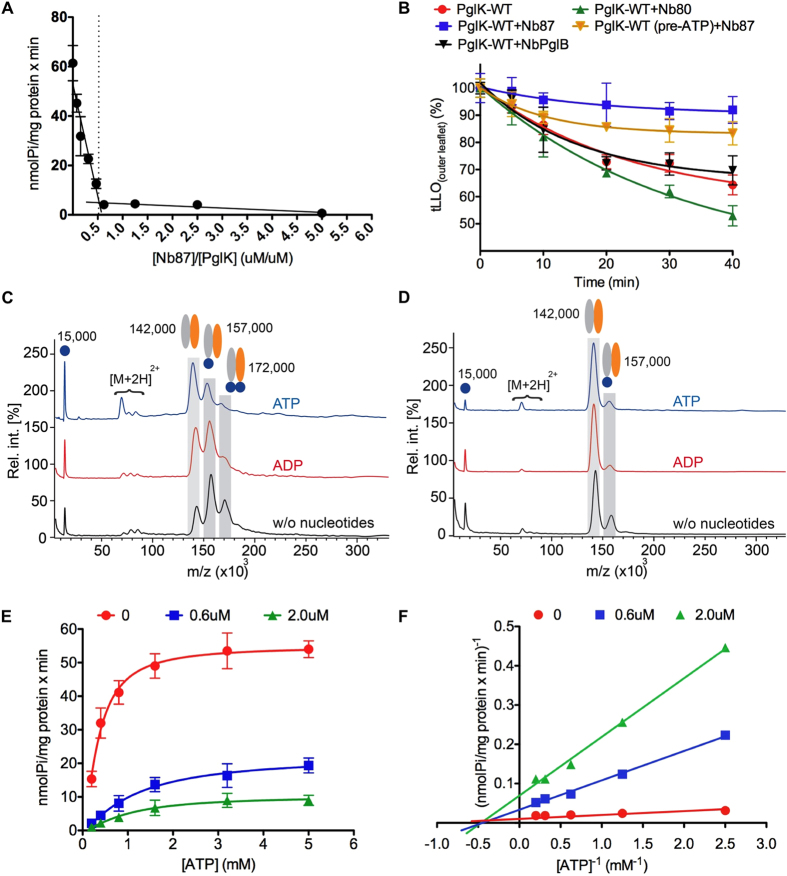
(**A)** ATPase activity of PglK in detergent (LMNG) in the presence of 0.08 to 5.0 molar equivalents of Nb87. The PglK concentration was kept constant at 1.6 μM, around two orders of magnitude above the expected Kd of nanobody binding^8^ (**B**) tLLO flipping in PglK-containing proteoliposomes in the presence of Nb87, a PglK-binding but non-inhibitory nanobody (Nb80), or a non-binding nanobody (NbPglB). “Pre-ATP” indicates a pre-incubation of PglK with ATP before the addition of Nb87. (**C** and **D**) High-mass MALDI mass spectra of cross-linked PglK–Nb complexes. Complex formation with Nb87 (**C**) or NbPglB (**D**) was investigated in the absence and presence of nucleotides. Unbound Nb ions were found around 15,000 m/z. Homo-dimeric PglK ions were detected around 142,000 m/z. Complex ions bound to one and two Nbs were found at 157,000 m/z and 172,000 m/z, respectively. [M+2 H]2+ corresponds to doubly charged complex ions. (**E)** ATPase activity of PglK as a function of ATP concentration in the presence of three different concentrations of Nb87. (**F)** Lineweaver-Burk plot to estimate the inhibitory modality of Nb87. All measurements were performed using a 1:2 PglK:Nb molar ratio in the presence of 5 mM nucleotides except for those showed in (**A** and **E)** Error bars denote s.d. (n = 3).

Remarkably, the Nb87-PglK complex structure shows a stoichiometry of one nanobody bound to one PglK homodimer. There is no significant asymmetry in PglK, suggesting that a second Nb87 copy could in principle be bound. In addition, a second Nb87 molecule would be fully compatible with the observed crystal lattice, with no clashes to prevent its binding. To validate the structural results and study the mechanism of inhibition, we have explored the PglK/Nb87 stoichiometry by determining the minimal molar equivalent of Nb87 necessary to reach maximum inhibition of the ATPase activity of PglK. (Fig. 3A). Upon titrating nanobody to PglK, the resulting ATPase activity plot reveals two lines that intersect at a 1:1 ratio of PglK-homodimer: Nb87, demonstrating that binding of a single nanobody to the PglK homodimer causes maximal inhibition. Furthermore, we used high-mass matrix-assisted laser desorption/ionization (HM-MALDI) mass spectrometry^26^ to qualitatively study the stoichiometry of the PglK-Nb87 complex that is present in solution. We incubated PglK and Nbs at a 1:2 molar ratio of PglK-homodimer:Nb in the absence of nucleotides or in the presence of 5 mM ATP or ADP. We subsequently cross-linked the formed complexes with low concentrations of glutaraldehyde^27^ (Fig. 3C and D). In the absence of nucleotides, 1:1 and 1:2 (PgIK-homodimer:Nb87) complexes are observed. However, the amount of 1:2 complexes decreases when nucleotide is present.

We have explored the mode of inhibition displayed by Nb87 (competitive, noncompetitive or uncompetitive) by measuring the ATPase rate of PglK as a function of ATP concentration in the presence of different amounts of Nb87 (Fig. 3E). The double-reciprocal (Lineweaver-Burk) plots derived from this data (Fig. 3F) show that Nb87 acts as a noncompetitive inhibitor, which implies that it displays affinity for both, the PglK-apo form and the PglK-nucleotide complex.

**Figure 4:**
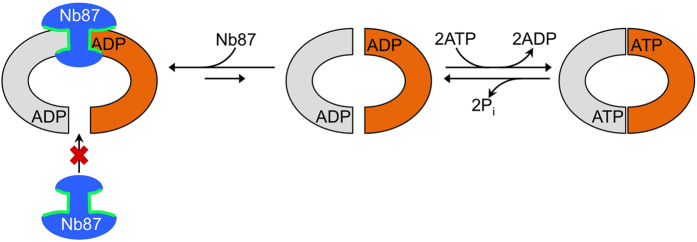
Schematic showing a cytoplasmic view of the “sticky-doorstop” inhibitory mechanism of Nb87. Orange and grey shapes depict the NBDs of PglK. The middle and right panels interconvert during productive ATPase and flippase cycles. PglK subunits are represented in colors grey and orange; Inhibitory Nb87 is represented in blue and its CDR loops in green.

These results confirm that even though Nb87 does not bind at or close to the molecular two-fold symmetry axis of PglK, the binding of one copy of the inhibitory Nb to PglK is preferred. Importantly, this happens not only *in crystallo* but also in solution. We therefore conclude that in the presence of nucleotides, once a first Nb87 molecule binds to PglK, a second Nb87 molecule is not able to access its binding epitope on the opposite side of the transporter. Thus, Nb87 acts as a “sticky doorstop” that decreases the chances for the PglK NBDs to close or open further (Fig. 4).

There are only few reported structures of ABC exporters bound to Nbs or antibodies: The structure of murine ABCB1 in complex with an inhibitory Nb showed a wide-open inward-facing conformation^17^. The Nb bound was located at the C-terminal end of one NBD, pointing towards the cytoplasm. This Nb strongly inhibited ABCB1 ATPase activity presumably by precluding the closing of NBDs, albeit in a different way than Nb87 due to the differences in the binding position and the fact that it interacts only with one NBD without restricting the conformation of ABCB1. Several cryo-electron microscopy structures of human ABCB1 bound to a Fab fragment from the inhibitory antibody UIC2 have been reported at ~15 Å resolution. These structures display a broad spectrum of inward facing states with different degrees of NBDs separation^18^. In this case, the UIC2-derived Fab fragment was bound to the extracellular region of ABCB1, and the antibody had no effect on the ATPase activity of ABCB1, but inhibits the efflux of certain drugs^28^,^29^. The inhibitory mechanism is not yet understood at molecular level, but it has been proposed that UIC2 is capable of restraining ABCB1 in specific conformations, preventing the completion of a full transport cycle^18^,^28^. In comparison to the ABCB1 cases, our structure reveals a new mode of inhibition of an ABC transporter by an antibody fragment.

### Implications for PglK mechanism

The structural and functional data presented here reveal that the two symmetrical ATPase sites of PglK are strongly coupled. Although the inhibitory nanobody only binds to one face of the transporter and directly inhibits hydrolysis at the closeby ATPase site, the second ATPase site, located some 30 Å away, is equally unable to hydrolyze ATP efficiently, suggesting strong conformational coupling. This allosteric effect is relevant because it is difficult to reconcile with fully independently operating ATPase sites in this symmetrical (homodimeric) ABC transporter. If our finding has general value, it would argue against a twin-engine ATPase activity of this transporter family^30^.

Combined with the functional data, our structural findings suggest that a transition to an inward-facing conformation is required for the inhibitory function of Nb87 to be triggered (Fig. 4). However, Nb87 inhibition is slowed when PglK is reconstituted in liposomes (Table 1 and Supplementary Figure 2B) or when PglK is pre-incubated with ATP before addition of Nb87 (Table 1). This would most likely suggest that under physiological conditions, wide-open inward-facing states might be less populated than in non-native conditions e.g. in detergent. This is in agreement with reduced inhibition of *in vitro* LLO flipping when PglK-containing proteoliposomes are pre-incubated with ATP (Fig. 3B and Supplementary Figure 2C), indicating that PglK can carry out several productive cycles of ATP hydrolysis and flipping, before the irreversible inhibition by binding of Nb87 occurs. In line with these findings, reduced cross-linking efficiency of PglK-Nb87 was observed by HM-MALDI analysis when PglK was incubated with ATP (Fig. 3C). These results suggest that in the native membrane, PglK will mostly adopt conformations with closed NBD dimers, as was speculated upon in our previous mechanistic model^6^.

We have previously described that outward-facing conformations are essential in the proposed LLO translocation mechanism^6^, as they define a hydrophilic translocation pathway for the LLO head-group. In this regard, the inhibitory effect of Nb87 on LLO flipping is a consequence of the impaired capacity of PglK to transform to outward-open conformations.

## Conclusions

The co-crystal structure of PglK with an inhibitory nanobody reveals a new, narrowly inward-facing conformation, providing the fourth snapshot in the conformational landscape of this ABC transporter. The inhibitory Nb87 appears to operate by a “sticky-doorstop” mechanism, whereby a single copy of the nanobody allosterically blocks the access to PglK for a second nanobody by locking a specific conformation of the homodimeric ABC transporter. By wedging between catalytic motifs at one of the two ATPase sites, Nb87 shuts down ATPase activity at both sites, probably by preventing NBD dimerization, and thus inhibiting LLO flippase activity. This mode of inhibition may apply to other ABC transporters. Our results further demonstrate that by trapping specific states, nanobodies are useful for structural and mechanistic studies of ABC transporters. Investigation of these types of inhibitory molecules is a promising area of biology, which may lead to design innovative strategies for the treatment of human affections^31^,^32^,^33^.

## Materials and Methods

### PglK expression and purification

The gene encoding PglK from *C. jejuni* was cloned into a modified pET-19b vector (Novagen) with a N-terminal His10 affinity tag and a TEV protease cleavage site^34^. PglK was overexpressed in *E. coli* BL21-Gold (DE3) (stratagene) cells, which were grown at 37 °C in Terrific Broth medium supplemented with 1% glucose (w/v) and induced with 0.2 mM IPTG. Cells were harvested by centrifugation, re-suspended in 50 mM Tris-HCl, pH 8.0; 500 mM NaCl; 7 mM β-mercaptoethanol; 0.5 mM PMSF and disrupted in a M-110 L microfluidizer (Microfluidics) at 15,000 p.s.i. chamber pressure. Membranes were pelleted by ultracentrifugation at 100,000 g for 0.5 h. PglK was solubilized in 50 mM Tris-HCl, pH 8.0; 500 mM NaCl; 20 mM Imidazole; 15% glycerol (v/v); 7 mM β-mercaptoethanol; 1% N-dodecyl-β-D-maltopyranoside (w/v) (DDM, Anatrace); 1% C_12_E_8_ anapoe (Anatrace) for 2 h. The supernatant was loaded onto a NiNTA superflow affinity column (Qiagen), washed once with the same buffer but containing 10% glycerol (v/v); 50 mM Imidazole; 0.02% DDM and then washed a second time with the same buffer containing 0.02% lauryl maltose neopentyl glycol (LMNG, affymetrix). Elution was performed in the same buffer containing 200 mM NaCl; 200 mM Imidazole. The protein was further purified by size exclusion chromatography (Superdex 200 10/300 GL, GE Healthcare).

### Reconstitution of PglK in proteoliposomes

Liposomes (20 mg lipid/ml) from a mixture 3:1 (w:w) of *E. coli* polar lipids and L-α-phosphatidilcholine (Avanti polar lipids) were prepared by extrusion through polycarbonate filters (400 nm pore size) and diluted in 10 mM Tris-HCl, pH 8.0; 150 mM NaCl. After saturation with Triton X-100, the liposomes were mixed with purified protein at a lipid/protein ratio of 50–30:1 (w/w). BioBeads were then added to remove detergent. Finally, proteoliposomes containing a final concentration of 20 mg/ml lipids, 6.2 μM PglK were centrifuged and washed before being frozen in liquid nitrogen and stored at −80 °C.

### Nanobodies production

To generate the nanobodies, 1 mg of PglK reconstituted in proteolipospomes was injected into a llama over a period of 6 weeks to elicit an immune response. The immunization, library construction, and nanobody selection was performed according to Pardon *et al*.^8^. Phage particles expressing nanobodies that bind to PglK were selected on solid phase coated PglK proteoliposomes. A clear enrichment was observed after two consecutive rounds of selection. 184 randomly chosen colonies - after the first and second round - were grown and induced to produce soluble Nanobody. Crude periplasmic extracts were screened in ELISA and the positive clones were sequence analyzed; 26 distinct families based on sequence alignments were discovered.

### Nanobodies expression and purification

Nbs were expressed in the *E. coli* WK6^8^. Bacteria were grown in terrific broth to an OD of 0.7 and then expression was induced by 1 mM IPTG overnight at 28 °C. Bacteria were then pelleted at 7,500 × g for 15 min at room temperature. Pellets were resuspended in TES buffer (0.2 M Tris at pH 8.0, 0.5 mM EDTA, and 0.5 M sucrose) and kept under slow agitation for 1 h at 4 °C. Two equivalents of four fold diluted TES buffer was added and kept under slow agitation for 1 h min at 4 °C. Samples were then centrifuged for 30 min at 4 °C and 8,000 rpm. Supernatant was used for purification on NiNTA resin (Qiagen). Binding to the Ni-NTA resin was performed at 4 °C for 1 h. The column was washed with 50 mM Tris pH 8.0, 250 mM NaCl, 20 mM Imidazole; then eluted with 50 mM Tris pH 8.0, 250 mM NaCl, 500 mM Imidazole. The protein was then desalted in buffer 20 mM Tris pH 8.0, 150 mM NaCl.

### PglK/N87 crystallization

We have purified the functional PglK mutant E510Q^6^, followed by incubation with purified Nb87 at molar ratios ranging from 1:1 to 1:3 PglK-homodimer:Nb87 in the presence of 5–10 mM Adenosine 5′-diphosphate (ADP) and 5–10 mM MgCl_2_. A monodisperse sample of the PglK-E510Q/Nb87 complex was isolated after size exclusion chromatography (Supplementary Figure 3) and concentrated to 8–10 mg/ml in an Amicon Ultra-15 concentrator (Millipore) with a molecular mass cutoff of 100 kDa. Additional ADP and MgCl_2_ were added before concentrating the sample. The protein was crystallized by vapor diffusion in sitting drops or hanging drops at 20 °C against reservoir containing 100–150 mM MOPS, pH 7.3; 50–150 mM NaCl; 28–32% PEG300. The protein to reservoir volume ratio was 2:1–1:1. Crystals typically appeared after 3–4 days and matured to full size within 2 weeks. Crystals were cryoprotected by gently increasing the cryoprotectant concentration in the drops (up to 30% PEG300) and directly flash frozen by immersion in liquid nitrogen before data collection.

### Data collection

Crystals belonged to the space group P22_1_2_1_ with unit cell dimensions: a = 84.34 Å, b = 142.66 Å, c = 199.48 Å. Diffraction data was collected at the beamline X06SA at the Swiss Light Source (SLS, Villigen). Data were processed and merged with XDS^35^ and anisotropic scaling/ellipsoid truncation was performed using the UCLA diffraction anisotropy server^36^. The resolution limits along a*, b* and c* were 4.0 Å, 3.9 Å and 3.9 Å respectively, which correspond to mild anisotropic data. To improve the usable resolution and quality of the resulting electron density maps, we used Karplus’ CC* (Pearson’s correlation coefficient) based data cutoff approach^37^. The resolution limit was set taking into account a CC_1/2_ > ~40% based on data merging statistics and a CC* analysis against unmerged intensities in Phenix package^38^ satisfying Karplus’ CC* against CC-work and CC-free criteria, as well as, R-free of the highest resolution shell against the refined structure being less than or equal to ~50%.

### Structures determination

The structure was determined by molecular replacement with a modified model of a PglK-E510Q structure determined previously at 2.9 Å (PDB code 5C78)^6^, and a homology model of Nb87 based on a 2.1 Å structure of another Nb with 81.7% identity (PDB code 4UU9) determined by another group (to be published). Molecular replacement was performed using the program Phaser^39^, refinement was performed using Phenix^38^ combined with manual building in Coot^40^. X-ray data and refinement statistics are given in Supplementary Table 1. The electron density map shown in Supplementary Figure 7A and B represent Fo-Fc polder OMIT maps^41^ calculated with Phenix^38^. The model coordinates of PglK-E510Q in complex with Nb87 were deposited at the Protein Data Bank with code 5NBD.

### PglH expression and purification

The gene encoding PglH was cloned into a modified pET-19b vector (Novagen) with a N-terminal His10 affinity tag fused to PglH. The protein was overexpressed in *E. coli* BL21-Gold (DE3) (stratagene) cells in Terrific Broth medium supplemented with 1% glucose (w/v). Cells were grown at 37 °C to A600 of 3.0 before the culture was induced by the addition of 0.5 mM IPTG and transfer to 18 °C for 16 h. All following steps were performed at 4 °C unless specified differently. Cells were harvested by centrifugation, re-suspended in 50 mM Tris-HCl, pH 8.0; 200 mM NaCl; 20 mM Imidazole; 0.5 mM PMSF and disrupted in a M-110L microfluidizer (Microfluidics) at 15,000 p.s.i. chamber pressure followed by addition of 1% TritonX-100 (w/v)^42^. After centrifugation the supernatant was loaded onto a NiNTA superflow affinity column (Qiagen), washed once with the same buffer but containing 50 mM Imidazole. Elution was performed in buffer containing 50 mM Tris-HCl, pH 8.0; 500 mM Imidazole. The protein was desalted into 50 mM Tris-HCl, pH 8.0; 150 mM NaCl.

### LLO and tLLO extraction

Isolation of LLOs was performed as described by Gerber *et al*.^43^. Briefly, LLOs were extracted from *E. coli* SCM6 cells carrying a *C. jejuni pglBmut* cluster, containing an inactivated *pglB* gene (LLO extraction) or a *pglHmut:pglBmut* cluster, containing an additionally inactivated *pglH* gene (tLLO extraction). Extraction was performed using a mixture of chloroform:MeOH:H_2_O, 10:10:3 (LLO) or MeOH:Chloroform 1:2 (tLLO). Extracts were dried in a rotavap and reconstituted in a buffer containing 10 mM Tris, pH 8.0, 150 mM NaCl, and 1% Triton X-100 (w/v). The concentration of reconstituted LLOs was determined by titrating various amounts of LLOs against a constant amount of acceptor peptide in an *in vitro* glycosylation assay as described before^44^.

### *In vitro* tLLO flipping assay

PglK proteoliposomes diluted in 10 mM Tris-HCl, pH 8.0; 150 mM NaCl were extruded through polycarbonate filters (400 nm pore size) and incubated with 5 mM MgCl_2_ and adenosine triphosphate (ATP) to initiate the tLLO flipping reaction, in the presence or absence of Nbs. To stop the translocation reaction, samples were diluted into a buffer containing 4 mM ADP. Labeling of non-flipped tLLO remnant in the external membrane leaflet of proteoliposomes was achieved after incubation with the glycosyltransferase PglH in the presence of 50 μM [^3^H]-UDP-GalNAc. To stop the labeling reaction the samples were filtered using Multiscreen vacuum manifold (MSFBN6B filter plate, Millipore) and washed with cold stop buffer. Radioactivity trapped on the filters was determined using a gamma counter (CobraII Auto-Gamma, Packard). Nonlinear fitting of data and initial velocities determination were performed using GraphPad Prism 5.

### ATPase assays

To study the effect of Nb binding on the ATPase activity of PglK, purified PglK was pre-incubated with each of the Nbs at a 1:2 molar ratio, and ATP hydrolysis rates were determined as described previously^6^, using a modified molybdate-based colorimetric method^45^. All reactions were performed in the presence of 5 mM ATP and 5 mM MgCl_2_. ATPase rates were determined using linear regression. Nonlinear regression and statistical analysis was performed using GraphPad Prism 5.

### Mutagenesis

PglK mutants were generated by either the QuickChange method or using gBlocks^®^ gene fragments (Integrated DNA technologies). The resulting plasmids of all constructs were validated by DNA sequencing (Microsynth). PglK variants were cloned into pMLBAD as above and used in flipping *in vivo* assays.

### Chemical Cross-linking and Sample Preparation

Chemical cross-linking is necessary to stabilize protein complexes for subsequent MALDI-MS analysis, and has been shown to not introduce any artifacts such as formation of nonspecific multimers^26^,^46^. Samples of PglK (18 μM) and Nbs (18 μM) in buffer containing 10 mM HEPES pH 7.5, 100 mM NaCl, 0.016% LMNG were incubated with 5 mM MgCl_2_, and either 5 mM ADP or 5 mMATP. After an incubation time of 10 minutes on ice, the cross-linking reaction was carried out using 0.1% (v/v) glutaraldehyde for 30 min. Unbound nanobodies and unreacted glutaraldehyde in the samples were removed using ultra-centrifugal filters (0.5 mL, NMWL of 100 kDa) from Merck Millipore (Germany). The samples were washed for 5 times with 100 μL buffer and were centrifuged at 8000 × g for 15 min at 4 °C.

### Mass Spectrometry

The samples were spotted on a stainless steel MALDI target plate using the sandwich spotting technique. Sinapic acid (10 mg/mL in acetonitrile/water/TFA, 49.95/49.95/0.1, v/v/v) was used as the MALDI matrix. All mass spectrometric measurements were performed in positive linear ion mode on a commercial MALDI-ToF/ToF mass spectrometer (ABI 4800, AB Sciex LLC, Framingham, MA USA) equipped with a high-mass detector (HM2, CovalX AG, Switzerland). The values for high voltage 1 and 2 of the HM2 detector were set to −3.6 kV and −20 kV, respectively. The ionization was induced with a Nd:YAG laser (355 nm) using 500 shots per spectrum. However, the best signal quality was obtained using a grid to source 1 ratio of 0.99 and a delay time of 1800 ns. Every spectrum was recorded in a mass range from 5000 to 1000000 m/z with a focused mass over charge value of 140000 m/z. The data obtained were smoothed using a second-order Savitzky-Golay smooth from Igor Pro (version 6.37, WaveMetrics, USA).

## Additional Information

**How to cite this article**: Perez, C. *et al*. Structural basis of inhibition of lipid-linked oligosaccharide flippase PglK by a conformational nanobody. *Sci. Rep.* **7**, 46641; doi: 10.1038/srep46641 (2017).

**Publisher's note:** Springer Nature remains neutral with regard to jurisdictional claims in published maps and institutional affiliations.

## Supplementary Material

Supplementary Information

## Figures and Tables

**Table 1:** PglK ATPase activity and Nbs effect.

	LMNG		Proteoliposomes	
	nmolPi/mg protein x min		nmolPi/mg protein x min	
	−LLO	+LLO	−LLO	+LLO
PglK-WT	58.2 +/− 2.1	104.8 +/− 8.3	58.3 +/− 4.5	118.9 +/− 15.4
PglK-WT + Nb80	57.1 +/− 3.4	97.6 +/− 9.6	62.3 +/− 5.1	104.5 +/− 12.3
PglK-WT + Nb87	4.1 +/− 1.7	6.3 +/− 3.2	11.3 +/− 3.5	21.4 +/− 9.2
PglK-WT + NbPglB	62.1 +/− 5.1	125.8 +/− 15.3	61.0 +/− 5.3	117.3 +/− 13.8
PglK-WT (pre-ATP) + Nb87	6.0 +/− 0.4	9.5 +/− 0.5	14.4 +/− 1.3	28.6 +/− 3.4
