# Supplementary material for: Structural basis of inhibition of lipid-linked oligosaccharide flippase PglK by a conformational nanobody
Source: Sci Rep. 2017 Apr 19;7:46641. doi: 10.1038/srep46641 (PMC5395944; doi:10.1038/srep46641)
Supplement: Supplementary Information [file srep46641-s1.pdf]

Supplementary information

Structural basis of inhibition of lipid-linked oligosaccharide flippase PglK by a conformational nanobody

Camilo Perez[1], Martin Köhler[2], Daniel Janser[1], Els Pardon[3,4], Jan Steyaert[3,4], Renato Zenobi[2], Kaspar P. Locher[1*]

[1]Department of Biology, Institute of Molecular Biology and Biophysics, ETH Zürich, CH-8093 Zürich, Switzerland

[2]Department of Chemistry and Applied Biosciences, ETH Zürich, CH-8093 Zürich, Switzerland

[3]VIB Center for Structural Biology, VIB, 1050 Brussels, Belgium

[4]Structural Biology Brussels, Vrije Universiteit Brussel, 1050 Brussels, Belgium.

[*]Correspondence and requests for materials should be addressed to K.P.L. (email: locher@mol.biol.ethz.ch).

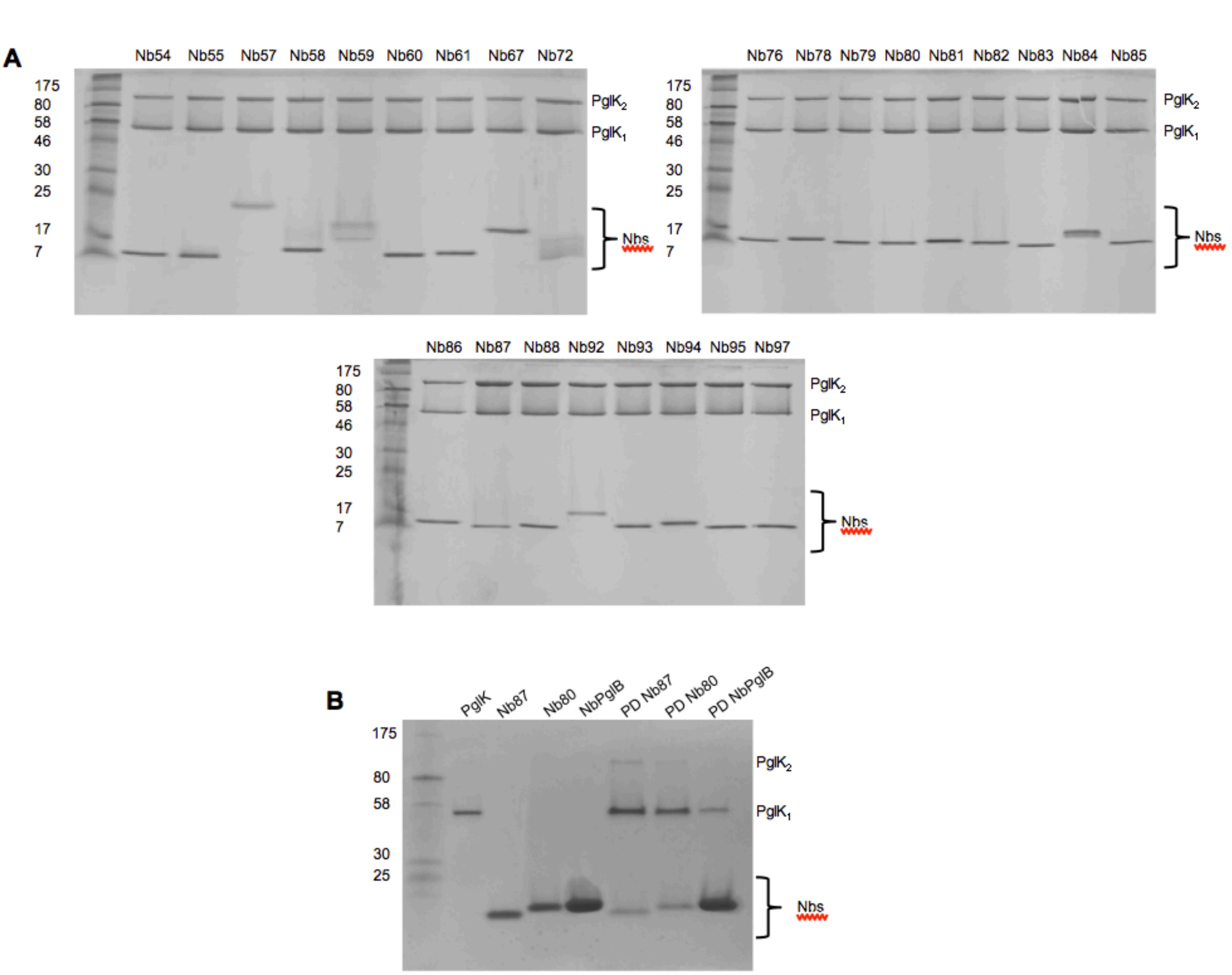

Supplementary Figure 1. A. SDS-PAGE of eluates from pull-down (PD) assays performed with Nbs representatives from each of the 26 distinct families. Purified, His-Tagged Nbs were immobilized using Ni-NTA resin, followed by passage of purified, tag-less PglK and extensive washing. Bound proteins were then eluted and resolved by SDS-PAGE. **B.** SDS-PAGE of purified PglK, Nb87, Nb80 and NbPglB together with PD assays performed with these purified proteins. NbPglB is a negative control. Although there is a light PglK band that co-eluted with NbPglB, the ratio of PglK:NbPglB is clearly much lower than the ratio with PglK specific Nbs after pull-down. $PglK_2$ and $PglK_1$ correspond to dimeric and monomeric PglK.

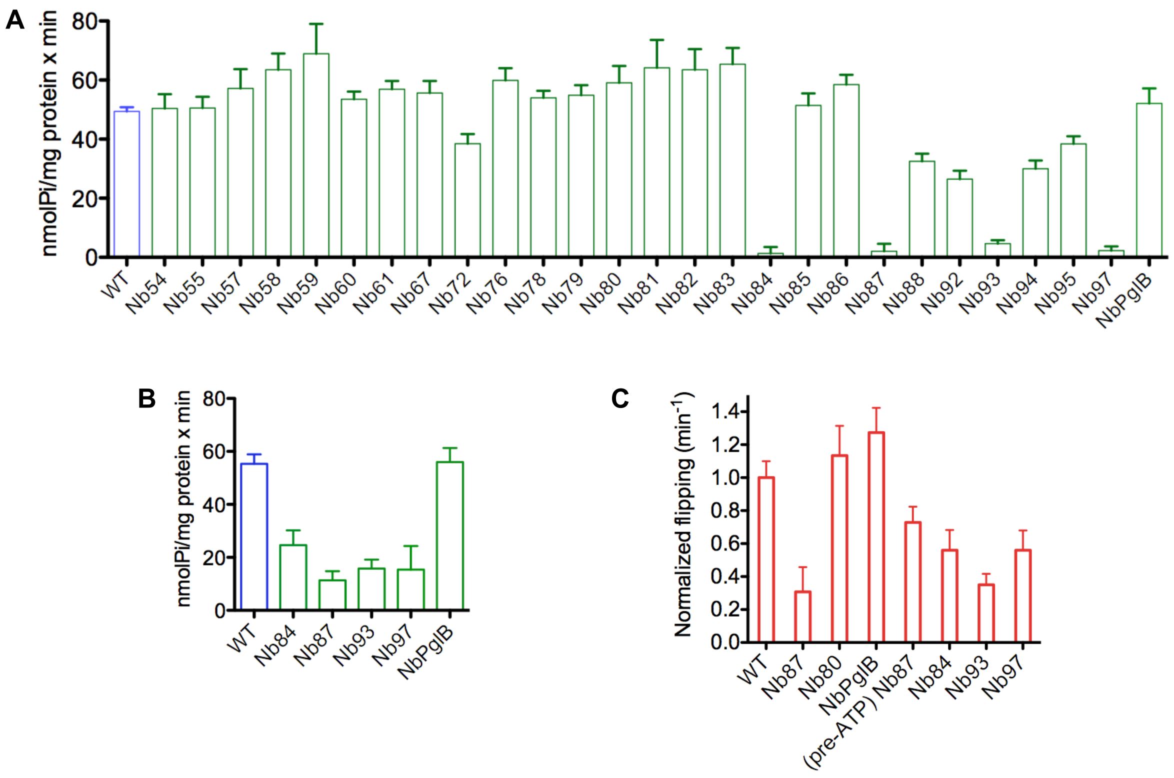

Supplementary Figure 2. ATPase activity assays of PglK in presence of Nbs in detergent (LMNG) (**A**) and proteoliposomes (**B**). **C.** tLLO flipping rates of PglK proteoliposomes in presence of inhibitory nanobodies (Nb84, Nb87, Nb93 and Nb97), positive control non-inhibitory nanobody (Nb80) and negative control non-inhibitory/non-binding nanobody (NbPglB). "Pre-ATP" indicate pre-incubation with ATP before addition of Nb87. All assays were performed using a 1:2 PglK:Nb molar ratio, in presence of 5mM ATP. Error bars denote s.d. (n=3).

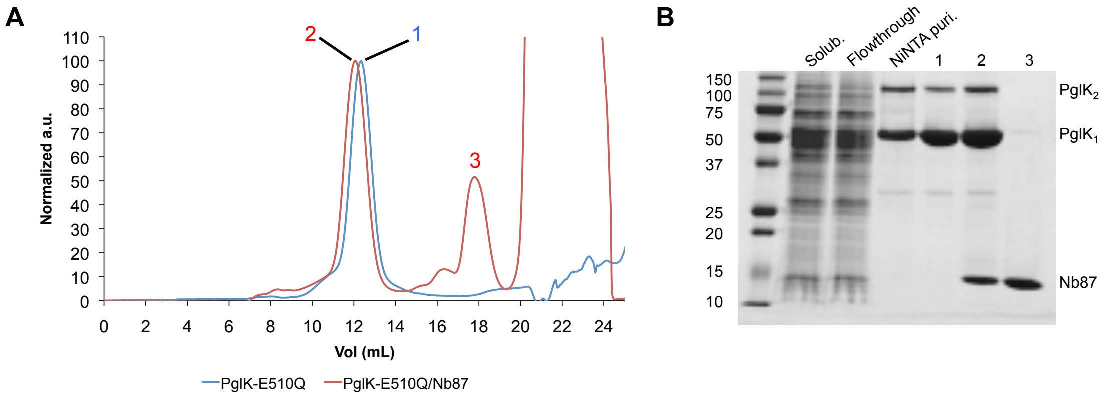

Supplementary Figure 3. PglK-E510Q/Nb87 complex isolation. **A.** Size exclusion chromatography (Superdex 200 10/300 GL, GE Healthcare) profiles of purified PglK-E510Q (blue curve) and PglK-E510Q pre-incubated with Nb87 at 1:2 PglK homo-dimer:Nb molar ratio (red curve). **B.** SDS-PAGE of samples from different steps of the experiment.

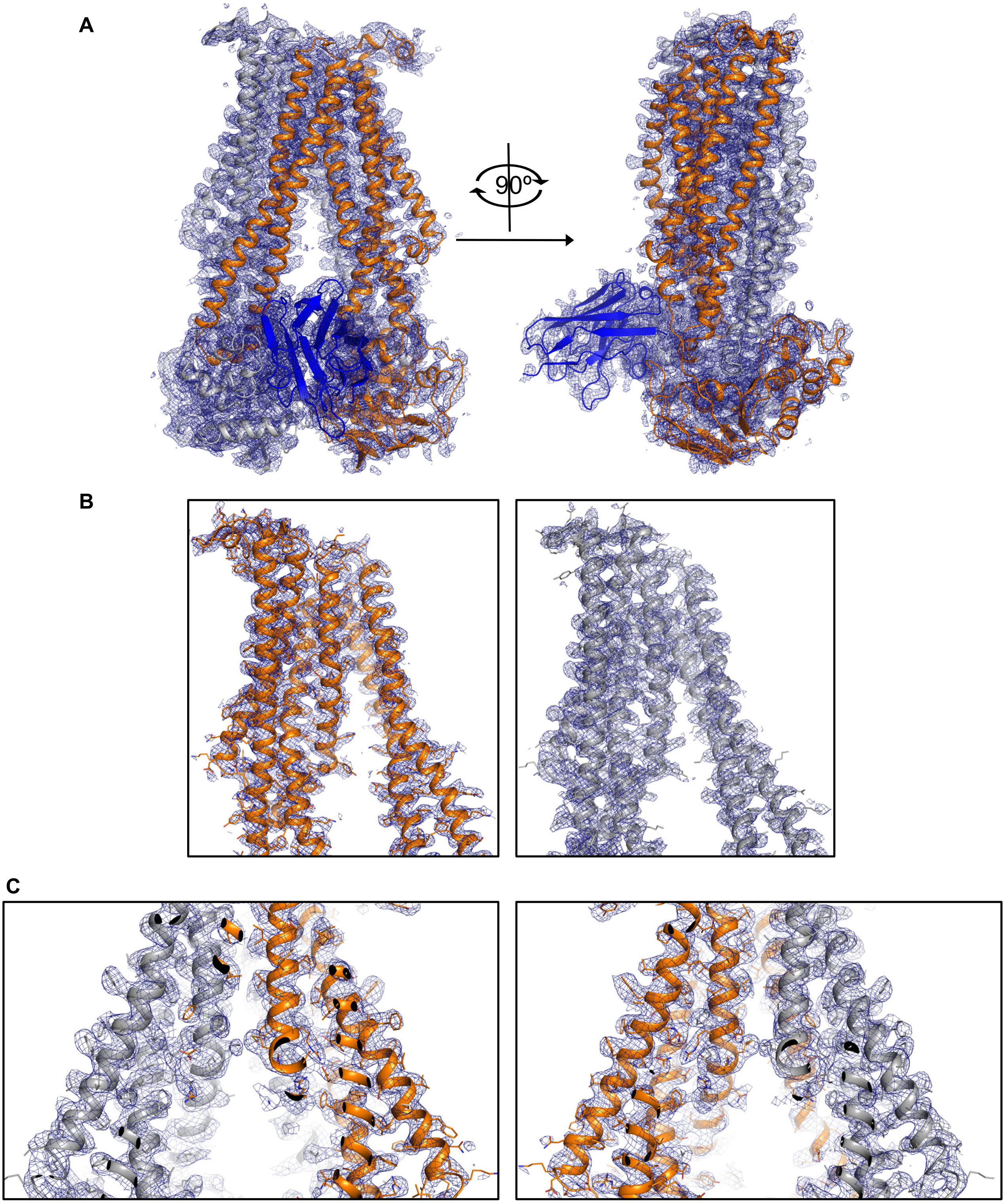

Supplementary Figure 4. 2Fo-Fc electron density map for the complete PglK-E510Q/Nb87 complex (**A**), transmembrane regions of individual subunits (**B**), and central cavity (**C**). All maps are shown at 1.0σ level.

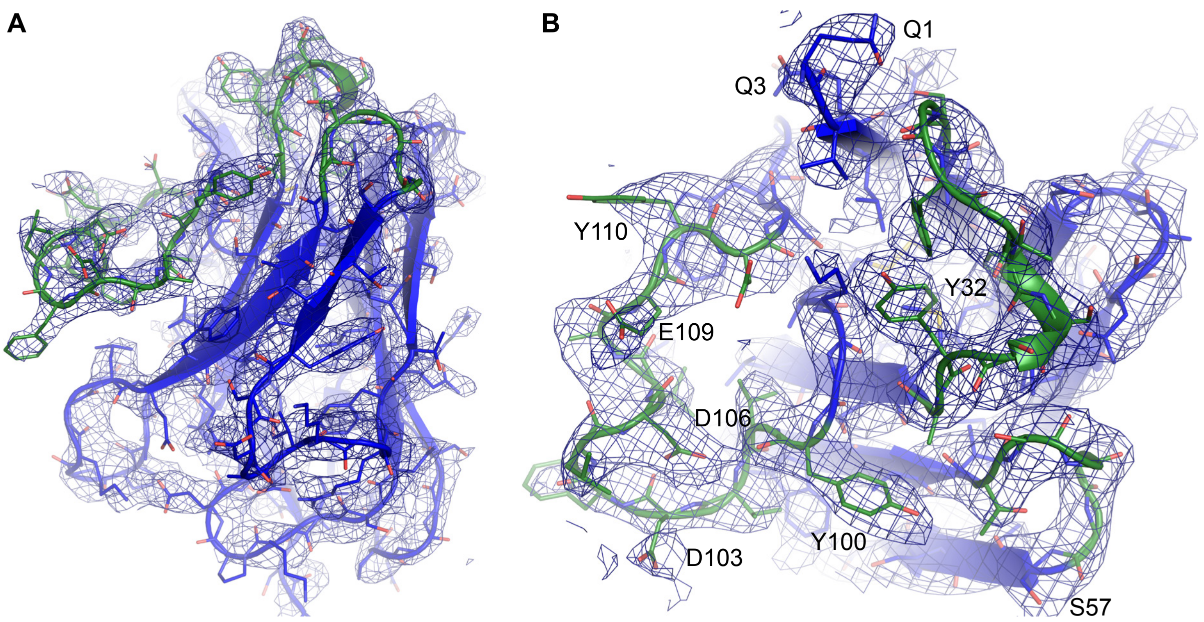

Supplementary Figure 5. Electron density map for Nb87. 2Fo-Fc electron density map of a side-view (**A**), and front-view (**B**) of Nb87 showing its CDR loops in green. All maps are shown at 1.0σ level.

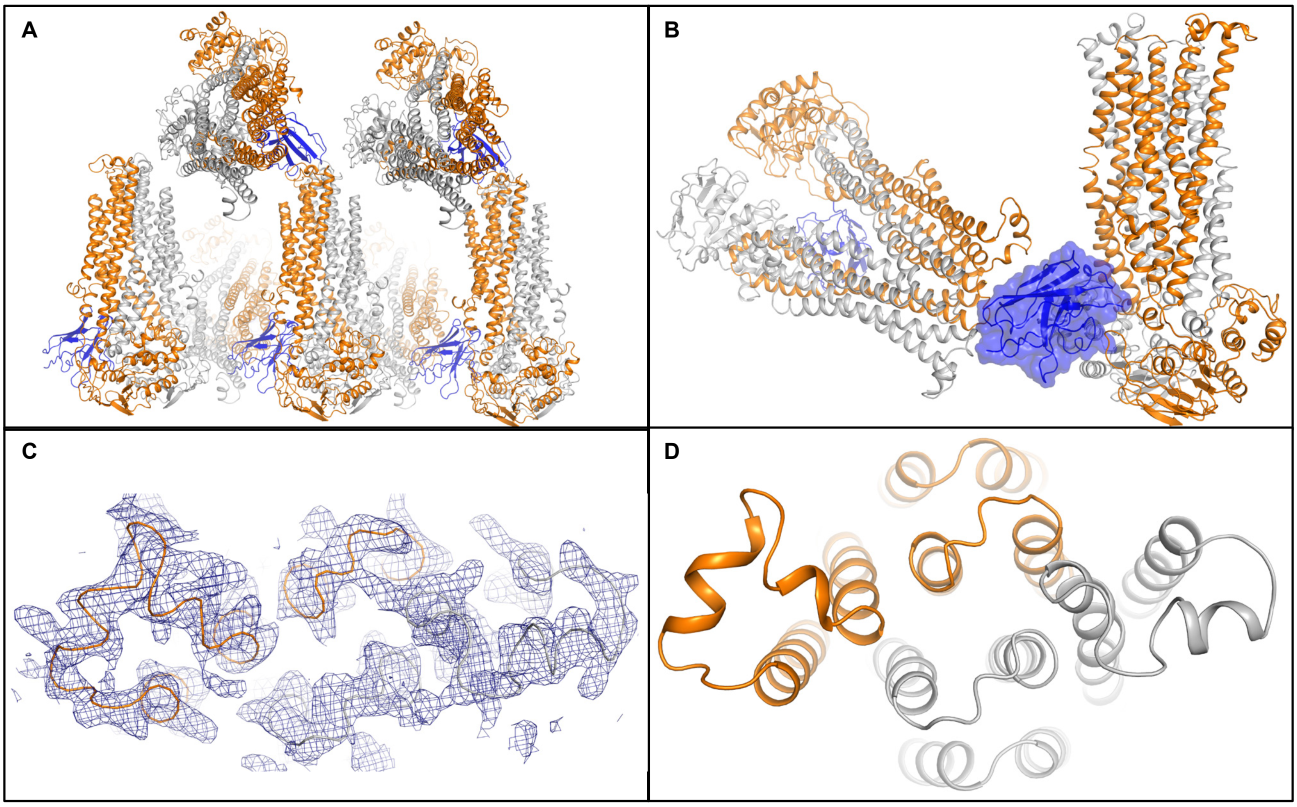

Supplementary Figure 6. Crystal packing of PglK-E510Q/Nb87 complex. Space group $P22_12_1$. Unit cell dimensions: a=84.34Å, b=142.66Å, c=199.48Å. **A.** Far view of crystal packing. **B.** Close up view showing crystal contacts formed by Nb87 (blue) and periplasmic loops from a neighbor PglK-E510Q molecule (Orange/Gray). **C.** and **D.** Top view of PglK-E510Q showing the 2Fo-Fc electron density map at 1.0σ level and the architecture of the external helices (EH). EH from the gray subunit is involved in crystal contacts formation and acquires a distinct conformation.

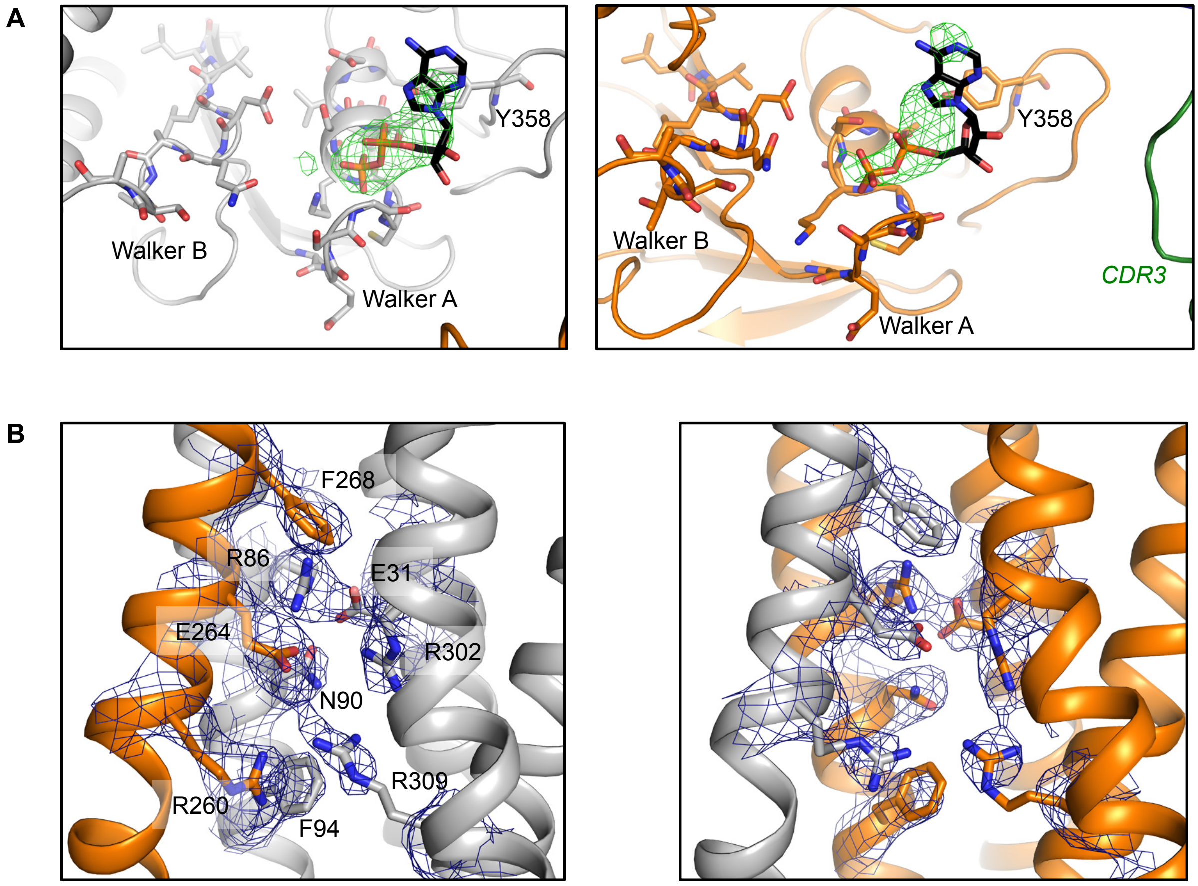

Supplementary Figure 7. A. Fo-Fc polder omit map shown at 3.0σ for the nucleotide binding sites in both PglK subunits. ADP molecules are shown in black with their pyrophosphate group in red-orange. PglK subunits are grey and orange; Inhibitory Nb87 is shown in blue and its CDR loops in green. **B.** Interactions of functionally important arginines in the central cavity. The 2Fo-Fc electron density map is shown at 1.0σ. PglK subunits are shown in grey and orange.

Supplementary Table 1: X-ray data collection and refinement statistics

**Data collection**	
Wavelength (Å)	1.0000
Space group	$P22_12_1$
Unit cell:	
a/b/c (Å)	84.34/142.66/199.48
α/β/γ (º)	90.0/90.0/90.0
Resolution (Å)	30-3.9
Completeness (%)	97.22[99.7]
No. measured reflections	299851 [307793]
No. unique reflections	41168 [42284]
I/σI	10.03(1.36) [9.87(0.9)]
R-merge (%)	10.9(207.0) [11.1(230.6)]
$CC_{1/2}$ (%)	100(40) [100(31)]
**Refinement**	
R_{work}/R_{free} (%)	31.1/33.5
R.m.s.d. Bonds (Å)	0.003
R.m.s.d. Angles (º)	0.607
Ramachandran plot:	
Outliers (%)	3.21
Allowed (%)	10.84
Favored (%)	85.94
Average *B*-factor (Å^2)	158.9
PDB ID	5NBD

Values in brackets are before anisotropic truncation
Values in parentheses are for the last resolution shell
Rmerge = $\Sigma_{hkl}\Sigma_i |I_i(hkl) - \langle I(hkl)\rangle| / \Sigma_{hkl}\Sigma_i I_i(hkl)$
